# Seasonal variations in the occurrence of acute appendicitis and their relationship with the presence of fecaliths in children

**DOI:** 10.1186/s12887-019-1824-9

**Published:** 2019-11-16

**Authors:** Yao-Jen Hsu, Yu-Wei Fu, Taiwai Chin

**Affiliations:** Changhua Children Christian Hospital, 135, Nan Hsiao Street, Changhua, Taiwan 500-06 Republic of China

**Keywords:** Appendicitis, Seasons, Fecalith

## Abstract

**Background:**

Acute appendicitis (AA) is the most common surgical condition in children. Although a higher incidence of AA in summer has been reported, the reason for this observation remains unclear. The purpose of this study was to compare the clinical findings of AA patients who underwent appendectomies during the summer months with those who underwent the procedure during the non-summer months.

**Methods:**

The clinical data of 171 patients who underwent appendectomy from January 2013 to December 2016 were reviewed. The patients were divided into a summer group (from May to October) and a non-summer group (from November to April) based on the month when appendectomy was performed. All patients were under 18 years of age at the time of surgery. The medical records including laboratory data, computed tomography scans, pathology reports and operative notes were reviewed.

**Results:**

The number of patients with AA was higher in the summer group than in the non-summer group (101 vs. 70 patients). No significant differences in the laboratory results between the two groups of patients were observed. The percentage of AA patients who presented with a fecalith was significantly lower in the summer group (33.6%) than in the non-summer group (55.7%). No significant differences in the incidence of appendiceal perforations and abscesses, as well as postoperative complications were observed between the two groups.

**Conclusions:**

The percentage of AA patients with fecaliths in summer was lower than that in the non-summer months. The increase in the number of AA patients in summer may be due to the increased occurrence of lymphoid hyperplasia, which may be correlated with the yearly outbreak of enterovirus infection during this period.

## Background

Acute appendicitis (AA) is a common surgical condition in children [1]. Complicated intra-abdominal infections in the paediatric population are generally caused by appendiceal perforations and are considered to be one of the most important causes of morbidity in children [2]. Laparoscopic appendectomy is a safe and effective procedure for the treatment of AA in children [3, 4]. Many studies have shown that the incidence of AA is higher in the summer [5–7]; however, the reason for this phenomenon remains unclear. Temperature, rainfall, atmospheric pressure [8], food [9] and dietary fibre [10, 11], air pollution [12], allergic reaction to pollen [6] and seasonal gastrointestinal infections [13] are factors contributing to the higher incidence of AA.

The purpose of the study is to provide additional evidence of this phenomenon by comparing the clinical information obtained from AA patients in summer with that obtained during the non-summer months.

## Methods

This retrospective study was approved by the Institutional Review Board of the hospital (IRB number 170710). We hypothesized the information obtained from the AA patients in summer will be different from that obtained from the patients in the non-summer months. Our primary goal is to find out the differences of AA in summer and non-summon months, and hopefully the seasonal pattern of the onset of AA can be explained. The secondary goal is to compare the postoperative complications in these two patient groups.

Patients under 18 years of age at the time of surgery who underwent appendectomies for AA in our institution from January 2013 to December 2016 were included in the study. Those who had an interval appendectomy or a pathological diagnosis of a normal appendix were excluded. The records of all included patients were reviewed retrospectively.

History taking, physical examination, image studies such as plain abdominal X ray, abdominal ultrasound or CT scan were performed in the emergency department if indicated. CT scan was not a routine examination for the diagnosis of AA in this children hospital, however, most of the patients already had CT scans when they were referred from other community hospitals. If a diagnosis of AA was confirmed, the patient underwent laparoscopic appendectomy with the technique which had been described in the literature [1].

Taiwan is situated in the subtropical zone with a relatively warm temperature. Details about the monthly variations in temperature in this country can be obtained from the Central Weather Bureau [14]. High temperatures (> 25 °C) are observed from May to October in middle Taiwan where our institution is located. The patients in the current study were divided into two groups (summer and non-summer) based on the month when the surgery was performed. The summer group included patients who were operated on between May and October, and the non-summer group included patients who underwent the surgical procedure between November and April. The following parameters were compared between the two groups of patients: age, gender, white blood cell counts, differential counts, C reactive protein (CRP) levels and the presence of a fecalith, appendicular perforation and an abscess. Postoperative complications such as wound infection, ileus duration longer than 5 days and postoperative intra-abdominal abscess were also compared.

Chi square and Student’s t-test were used for statistical evaluation. The significance level was set at *p* < 0.05.

## Results

One hundred and seventy-one patients had appendectomies during the study period; among them, 160 patients had undergone preoperative computed tomography (CT) scans. Pathology reports were obtained from all 171 patients. Fecaliths were identified in the CT scans of 34 patients and reported in the pathology reports of 18 patients; in 21 patients, information about the presence of a fecalith was obtained from both the CT scan and the pathology report. The summer and non-summer groups comprised 101 and 70 patients, respectively. In the summer group, 34 patients presented with a fecalith and 26 had perforation and/or an abscess. In the non-summer group, 39 patients had a fecalith, and 25 patients had perforation and/or an abscess (Table 1).

**Table 1:** Patient data during the summer and non-summer months

	Summer	Non-summer	p
Patient number	101	70	
Age (year) (mean ± SD)	12.2 ± 3.7	11.4 ± 4.2	0.290 ^b^
Male: female	70:31	45:25	0.363 ^a^
AA with fecalith (n(%))	34 (33.6%)	39 (55.7%)	0.004 ^a^
Perforation/abscess (n(%))	26 (25.7%)	25 (35.7%)	0.161 ^a^
CRP (mg/dL) (mean ± SD)	6.64 ± 7.78	9.06 ± 9.61	0.087^b^
WBC*1000/μL (mean ± SD)	16.1 ± 5.0	15.4 ± 4.9	0.392^b^
Neutrophils (%)(mean ± SD)	81.46 ± 9.67	81.57 ± 7.96	0.938^b^
Lymphocytes (%)(mean ± SD)	10.97 ± 7.47	10.44 ± 6.59	0.643^b^
Monocytes (%)(mean ± SD)	6.53 ± 2.73	7.23 ± 2.80	0.115^b^
Hospital stay (days±SD)	5.00 ± 3.32	5.59 ± 3.51	0.304^b^
Wound infection (n(%))	1 (0.99%)	1 (1.43%)	0.645 ^a^
Postop ileus> 5 days (n(%))	2 (1.98%)	3 (4.29%)	0.676 ^a^
Postop abscess (n(%))	1 (0.99%)	2 (2.86%)	0.747 ^a^

^a^Chi-square test; ^b^ t-test

No significant differences in age and gender were observed between the summer and non-summer groups. The average CRP level was higher in the non-summer group, but the difference was not statistically significant when compared with the summer group (Table 1). Likewise, no significant differences in other laboratory results were noted between the two groups.

**Fig. 1:**
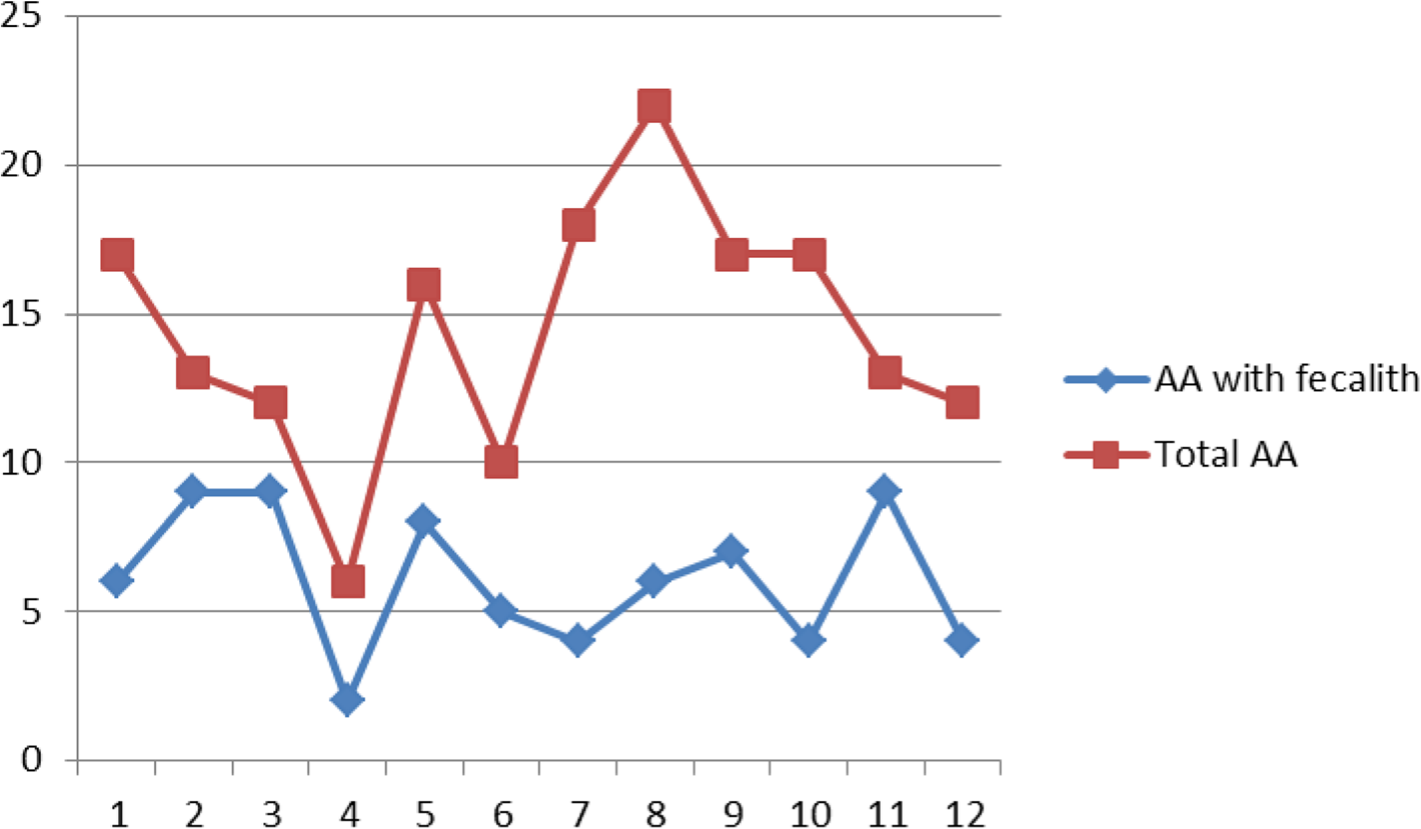
The total number of AA patients and those who presented with a fecalith each month. The total number of AA cases was higher in summer, but the number of AA patients with a fecalith was relatively the same throughout the year

Details about the total number of patients with AA and those who presented with a fecalith each month are shown in Fig. 1. The total number of AA patients increased in the summer, mainly due to the increase in the number of patients who did not present with a fecalith during summer. The number of patients with a fecalith remained relatively stable throughout the year. The percentage of AA patients who presented with a fecalith was significantly higher (*p* = 0.004) in the non-summer group (55.7%) than in the summer group (33.6%; Table 2).

**Table 2:** The number of patients with fecaliths and perforations in the summer and non-summer groups

	Summer	Non-summer	Total	p
Fecalith(n(%))	34 (33.6%)	39 (55.7%)	73 (42.7%)	0.004^a^
No fecalith (n(%))	67 (66.4%)	31 (44.3%)	98 (57.3%)	
Perforation/abscess (n(%))	26 (25.7%)	25 (35.7%)	51 (29.8%)	0.161^a^
No perforation/abscess (n(%))	75 (74.3%)	45 (64.3%)	120 (70.2%)	

^a^Chi-square test

The presence of a fecalith was associated with the presence of an appendicular perforation or abscess (*p* = 0.001; Table 3). However, the incidence of perforation and/or an abscess was not significantly different between the summer and the non-summer groups (*p* = 0.161; Table 2).

**Table 3:** The correlation of fecaliths and appendiceal perforation

	Fecalith	No fecalith	Total	p
Perforation/abscess (n(%))	34 (66.7%)	17 (33.3%)	51	0.001^a^
No perforation/abscess (n(%))	39 (32.5%)	81 (67.5%)	120	

^a^Chi-square test

The average hospital stay of the non-summer group was longer than the summer group. The percentages of the patients who had wound infection, ileus and postoperative intra-abdominal abscess were also higher in the non-summer group. However, the differences of the hospital stay, wound infection, ileus and postoperative intra-abdominal abscess between the two groups were not statistically significant (Table 1).

## Discussion

Similar to previous studies [13, 15], an increase in the number of patients with AA during summer was observed in the current study. Further analysis showed that this phenomenon was mainly due to the increase in the number of AA patients who presented without a fecalith. The appendix is a blind-ended structure; hence, the obstruction of its outlet will lead to the accumulation of both mucus and bacterial flora resulting in acute inflammation. Most surgeons agree that fecaliths can obstruct the lumen of the appendix. Although lymphoid hyperplasia may play a role in the obstruction of the appendiceal lumen in the absence of a fecalith, its role in AA is not well established.

**Fig. 2:**
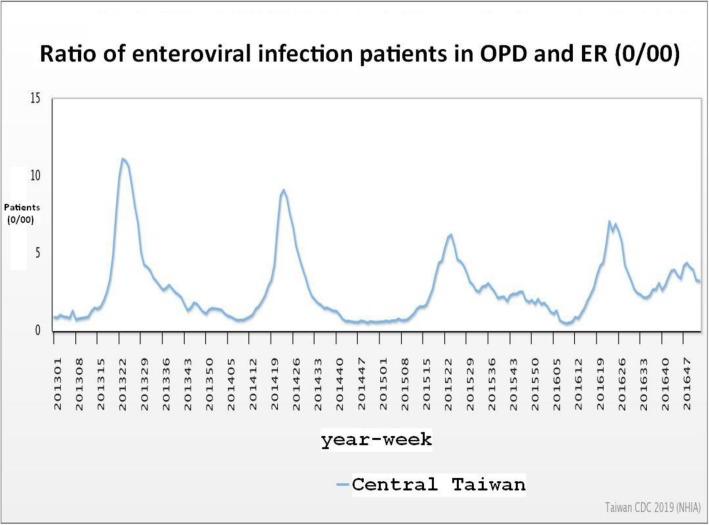
The ratio (0/00) of patients with a diagnosis of enterovirus infection (open source from Taiwan National Infectious Disease Statistics System, Taiwan Centers for Disease Control)

Lymphoid hyperplasia can occur during a viral outbreak, which could increase the probability of an appendiceal lumen obstruction. In an epidemiologic study by Alder et al. (2010), a correlation between influenza and AA was reported [16]. However, influenza is more common in winter. The Taiwan Centers for Disease Control reported a yearly outbreak of enterovirus infection from May to October in recent years [17] (Fig. 2). Furthermore, enterovirus is the only virus that causes an obvious outbreak in summer (May to October). These findings suggest that the rise of AA in summer may be correlated with this viral outbreak.

The association between the presence of a fecalith and an appendiceal perforation had been reported previously; Alaedeen et al. (2008) showed that the appendix was perforated in 57% of patients who had a fecalith when compared with 36% of patients who did not present with a fecalith [18]. A similar association was found among the patients in the current study. A higher incidence of perforations was noted in the non-summer group than in the summer group, but the difference did not reach statistical significance.

There are some limitations to this study. Firstly, this is a retrospective, single-institution study with a limited number of patients. Secondly, there is no direct evidence of the aetiology of AA. Nevertheless, to the best of our knowledge, the increase in the incidence of AA in the absence of a fecalith, particularly in summer, has not been reported in the literature so far.

## Conclusions

This study shows the differences in the clinical information between the summer and non-summer groups of AA patients. The increase in the number of AA patients in the summer may be due to the occurrence of lymphoid hyperplasia, which may be related to the yearly outbreak of enterovirus infection in this region during that period. Further studies involving multiple institutions and larger sample sizes are needed to confirm the correlation.

## Data Availability

The datasets used and analysed during the current study are available from the corresponding author on reasonable request.

## Abbreviation

AA: Acute appendicitis
